# Fusing graph transformer with multi-aggregate GCN for enhanced drug–disease associations prediction

**DOI:** 10.1186/s12859-024-05705-w

**Published:** 2024-02-20

**Authors:** Shihui He, Lijun Yun, Haicheng Yi

**Affiliations:** 1 School of Information Science and Technology, Yunnan Normal University, Kunming, 650500 China; 2 Engineering Research Center of Computer Vision and Intelligent Control Technology, Department of Education, Kunming, 650500 China; 3 School of Computer Science, Northwestern Polytechnical University, Xi’an, 710129 China

**Keywords:** Drug repositioning, Drug–disease associations, Graph transformer, Graph neural networks, Neural collaborative filtering

## Abstract

**Background:**

Identification of potential drug–disease associations is important for both the discovery of new indications for drugs and for the reduction of unknown adverse drug reactions. Exploring the potential links between drugs and diseases is crucial for advancing biomedical research and improving healthcare. While advanced computational techniques play a vital role in revealing the connections between drugs and diseases, current research still faces challenges in the process of mining potential relationships between drugs and diseases using heterogeneous network data.

**Results:**

In this study, we propose a learning framework for fusing Graph Transformer Networks and multi-aggregate graph convolutional network to learn efficient heterogenous information graph representations for drug–disease association prediction, termed WMAGT. This method extensively harnesses the capabilities of a robust graph transformer, effectively modeling the local and global interactions of nodes by integrating a graph convolutional network and a graph transformer with self-attention mechanisms in its encoder. We first integrate drug–drug, drug–disease, and disease–disease networks to construct heterogeneous information graph. Multi-aggregate graph convolutional network and graph transformer are then used in conjunction with neural collaborative filtering module to integrate information from different domains into highly effective feature representation.

**Conclusions:**

Rigorous cross-validation, ablation studies examined the robustness and effectiveness of the proposed method. Experimental results demonstrate that WMAGT outperforms other state-of-the-art methods in accurate drug–disease association prediction, which is beneficial for drug repositioning and drug safety research.

## Background

Identification and characterization of potential interactions between drugs and diseases are crucial challenges for drug discovery and disease treatment. Conventional methods for validating drug–disease associations rely on costly and time-consuming experimental procedures. The average cost of bringing a new drug to market exceeds about 2 billion dollars and 10–15 years before it can reach the pharmacy shelf [1–3]. Therefore, finding new indications for existing drugs, also known as drug repositioning, is an economically viable and time-saving strategy [4, 5]. Computational methods for drug repositioning facilitate the identification of potential drug–disease associations by screening large-scale data sources, enabling more rational design of clinical trials. Such strategies can accelerate the drug discovery pipeline and increase the availability of new treatments [6].

The utilization of computational methods for drug repositioning in drug discovery has become widespread [7, 8]. During this time, a growing array of methodologies has emerged. For example, methods rooted in matrix decomposition, like the one by Cui et al. [9] utilize dual-network L_2,1_-collaborative matrix factorization for predicting novel drug–disease interactions. Fu et al. [10] introduced MFLDA, a method that decomposes heterogeneous data sources' matrices into low-rank forms using matrix tri-factorization, thus exploring and exploiting their inherent and shared structure. MFLDA facilitates the selection and integration of these data sources by assigning varying weights to each source. Matrix factorization diminishes data dimensionality by transforming matrices into low-rank structures, extracting crucial features and patterns. However, with extensive or densely high-dimensional matrices, its computational demands might become excessive, resulting in reduced efficacy in managing considerable noise.

Network-based drug repositioning models have emerged to confront this challenge, striving to capitalize on intricate relational networks among biological entities such as drugs and diseases. These models amalgamate varied information sources, encompassing protein–protein interaction networks, gene expression data, and drug compound information, to anticipate potential novel drug–disease associations. For instance, Zhang et al. [11] proposed NTSIM to predict unobserved drug–disease associations and extended it to NTSIM-C for classifying therapeutic associations. Zhang et al. [12] proposed SCMFDD, projecting drug–disease associations into two low-rank spaces, revealing latent features, and introducing feature-based similarity and semantic constraints. Lu et al. [13] proposed heterogeneous information network (HIN) based model, namely HINGRL. Zhou et al. [14] introduced NEDD, using varied-length metapaths to explicitly capture internal relationships within drugs and diseases and obtain low-dimensional representation vectors. Martínez et al. [15] developed DrugNet, a network-based method predicting new drug uses and treatments for diseases. It utilizes a heterogeneous network formed from disease, drug, and target information, identifying novel associations by information propagation.

Despite the commendable interpretability inherent in network-based methodologies, their performance is deemed unsatisfactory. However, the drug–disease association network naturally has a graph structure, which enables techniques that leverage graph neural networks to adeptly preserve essential information, eliminate noise, and extract pivotal patterns and features. Therefore, this enhances the accuracy of information available for prediction and analysis in graph-based data scenarios. Some methods have been proposed to exploit the advantages of graph neural networks for drug–disease association prediction. Yu et al. [16] proposed LAGCN, a method that integrates heterogeneous networks, employs graph convolutional operations, and incorporates an attention mechanism. Yang et al. [17] introduced a model that infers drug–disease associations by applying network-embedding algorithms alongside a random forest classification approach. Gu et al. [18] introduced REDDA, a heterogeneous graph neural network with three attention mechanisms for sequential drug disease representation learning. Wu et al. [19] proposed EMP-SVD, a novel framework that predicts drug–disease associations by integrating multiple meta-paths and singular value decomposition. Li et al. [20]. proposed the NIMCGCN method, which integrates Graph Convolutional Networks (GCN) with Neural Inductive Matrix Completion (NIMC) models to discover associations between miRNA and diseases. VGAE [21]. introduced by Kipf et al., is a graph neural network model based on the Variational Autoencoder (VAE) framework, which models node features through an encoder-decoder structure, mapping them to a latent space distribution. Meng et al. [22] leveraged deep learning within a heterogeneous network framework to identify potential drugs related to diseases. Their model, DRWBNCF, employs weighted bilinear graph convolution operations, intricately fusing information about drug–disease associations and drug–disease similarity networks. Tang et al. [23] proposed the DRGBCN model, which exploits the embedding of graph convolutional layers and local interactions between drugs and diseases, thus significantly improving the accuracy and reliability of predictions. Ghasemian et al. [24] applied meta-learning methods in network analysis to develop a stacked model that integrates complex prediction algorithms from various domains, effectively mitigating changes in link prediction. Additionally, many studies [25, 26] have indicated that collaborative drug combination prediction is widely applied in drug repositioning. For instance, the SNRMPACDC model proposed by Li et al. [27] combines Siamese convolutional networks and random matrix projection to predict collaborative combinations of anticancer drugs. NLLSS [28] is a semi-supervised learning-based model that focuses on predicting collaborative drug combinations, enhancing the model's predictive performance through methods involving non-negative low-rank and sparse structures. They play a crucial role in revealing the associations between drugs and biomolecules, as well as in drug repositioning.

**Fig. 1:**
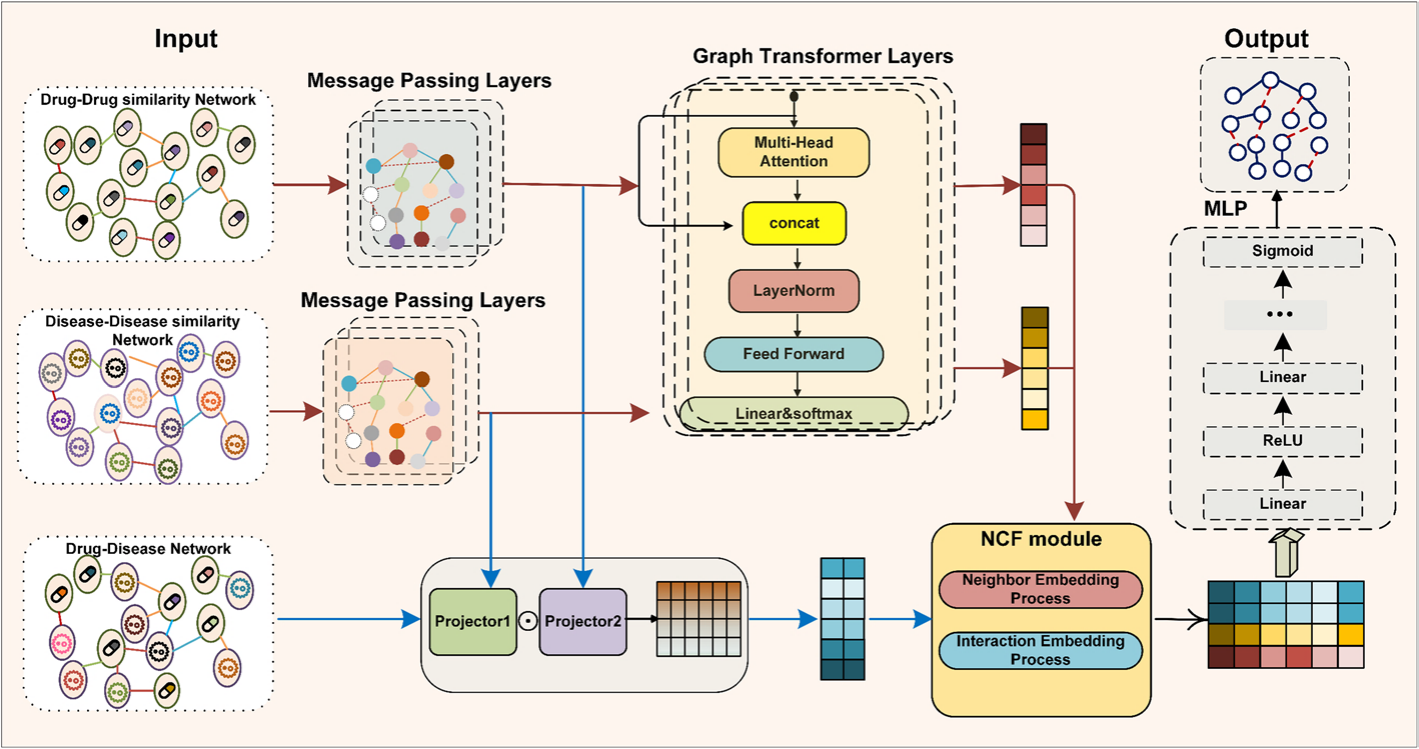
The overall architecture of the proposed WMAGT. WMAGT involves three main steps. First, drug and disease similarity networks are jointly encoded using GCN and graph transformer for representation projection. In the second step, matrix operations project drug and disease representations in the network, generating new information. Lastly, the domain information from the first step and interactive information from the second step are utilized in the NCF module, and multiple loss functions along with MLP are employed to comprehensively model the drug–disease relationship

Although these methods can extract edge information through extensive informatics learning, they often struggle to fully mine the complex interactions between nodes in heterogeneous graphs, which may affect the accuracy of predictions. To address these challenges, we propose a heterogeneous information graph representation learning method for predicting drug–disease associations, named WMAGT, which utilizes weighted multi-aggregate graph convolutional network and graph transformer to exploit discriminative node representations. The workflow of the proposed method is demonstrated in Fig. 1. Specifically, the proposed WMAGT approach first integrates drug–drug similarity networks, disease–disease similarity networks, and validated drug–disease association networks to construct a comprehensive heterogeneous information network. Then, graph transformer combined with weighted multi-aggregate graph convolutional neural network are used to learn efficient characterizations of drugs and diseases from this heterogeneous information network. Prior to the predictor, we integrated domain embeddings and interaction embeddings through neural collaborative filtering for the final link prediction scoring. To evaluate the performance of the proposed method, we cross-validated the predictive performance of WMAGT on three benchmark datasets and compared it with four state-of-the-art methods while conducting ablation experiments. Experimental results provide strong evidence of the effectiveness of WMAGT in discovering drug indications, which is important for advancing drug repurposing and reducing adverse drug reactions in the field of drug discovery. The main contributions and advantages of WMAGT include:

1. Proposing a representation learning method based on heterogeneous information graphs, which fully utilizes the multi-source information of drugs and diseases and considers their multi-level relationships.
2. Adopting a representation learning framework of Graph Transformer and Weighted Multi-Aggregation Graph Convolutional Neural Network, effectively eliminating the impact of heterogeneity and capturing relationships to learn more effective node representations.
3. Demonstrating through experiments on three public datasets that this method has broad application prospects in the field of drug discovery. Our approach not only outperforms existing models in predictive performance but also shows significant improvement in understanding complex biological networks.

## Methods

In this study, we propose a novel computational method, WMAGT, for drug repositioning, aiming to discover new indications for existing drugs by inferring potential drug–disease associations. First, we build a heterogeneous network that incorporates various types of relations in the data set, such as drug–drug similarity network, disease–disease similarity network, and drug–disease association network. Then, we utilize an end-to-end model to learn the latent features of the network and predict the unknown associations.

### Benchmark datasets

To explore heterogeneous network prediction methods for drug–disease associations, we utilized three publicly available real datasets to assess the efficacy of our model. The first dataset, Fdataset [29], comprises 313 diseases from the OMIM database [30] and 553 drugs from the DrugBank database [31], along with 1933 known associations between them. Another dataset, termed as Cdataset [32], consists of 663 drugs from the DrugBank database and 409 diseases from the OMIM database, encompassing 2532 established associations between drugs and diseases. The third dataset is LRSSL [33], which comprises 763 drugs from the DrugBank database, 681 diseases from the MeSH database, and a collection of 3051 validated associations between drugs and diseases. The essential statistical information of these three datasets is presented in Table 1.

**Table 1:** Details of the three benchmark datasets

Datasets	Drugs	Diseases	Associations
Fdataset	593	313	1933
Cdataset	663	409	2532
LRSSL	763	681	3051

### The construction of heterogeneous information graph

To predict potential drug–disease associations, this research employed network analysis methods based on a known drug–disease association network denoted as *G*. *G* is represented by an *n* × *m* binary matrix *A*, where *n* and m represent the number of drugs and diseases, respectively. The matrix *A*_*ij*_ holds a value of 1 or 0, indicating the presence or absence of an experimentally validated association between drug *r*_*i*_ and disease *d*_*j*_.

Two additional similarity networks were constructed: a drug–drug similarity network *G*_*r*_ and a disease–disease similarity network *G*_*d*_. These networks are represented by *n* × *n* and *m* × *m* matrices *A*_*r*_ and *A*_*d*_, respectively. The values *A*_*r*_(*i*, *j*) and *A*_*d*_(*i*, *j*) represent the similarities between drug *r*_*i*_ and drug *r*_*j*_, and between disease *d*_*i*_ and disease *d*_*j*_, respectively. These similarities were computed based on various characteristics including chemical, pharmacological, therapeutic, phenotypic, genetic, and environmental properties of drugs or diseases.

To enhance accuracy and reduce noise, a *k*-nearest neighbor approach was employed. It considered only the *k* most similar neighbors for each drug or disease. The extended *k*-nearest neighbor sets of drugs or diseases, represented as $\tilde{N}_{k}$, comprised the individual entities along with their *k* nearest neighbors. *A*_*r*_(*i*, *j*) and *A*_*d*_(*i*, *j*) illustrate the similarities among drugs or diseases, considering their extended k-nearest neighbor sets $\tilde{N}_{k}$. Mathematical representation:

1 $$G = (A_{ij} )_{n \times m}$$

2 $$G_{r} = (A_{r} \left( {i,j} \right))_{n \times n}$$

3 $$G_{d} = (A_{d} \left( {i,j} \right))_{m \times m}$$

Considering two sets representing drugs (*R*) and diseases (*D*), where each $r \in R$ and $d \in D$ introduces an association label *Y*_*r*,*d*_ signifying the presence *Y*_*r*,*d*_ = 1 or absence *Y*_*r*,*d*_ = 0 of an association between drug r and disease *d*. Consequently, inferring the association label *Y*_*r*,*d*_ for a given drug r and disease d relies on known associations within the sets. The expression for the association label *Y*_*r*,*d*_ remains defined as:

4 $$Y_{r,d} = \left\{ {\begin{array}{*{20}l} 1 \hfill & {\quad if\,r\,is\,associated\,with\,d} \hfill \\ 0 \hfill & {\quad otherwise} \hfill \\ \end{array} } \right.$$

This representation aims to establish the foundation of the drug repositioning problem, framing it as a task of predicting association labels.

### Graph convolutional network module

In a drug–disease heterogenous graph, nodes represent various drugs and diseases. Typically, each node contains its own similarity information, and the edges connecting two nodes represent the relationship between them. We employ Graph Convolutional Networks (GCN) [34–37] to integrate node information, which usually consists of aggregation functions and update functions. Aggregation functions are applied to each node/edge to gather information from their neighbors, while update functions generate new representations for each node/edge based on the collected information and the previous representation. The update function is defined as follows:

5 $$H^{{\left( {l + 1} \right)}} = \sigma \left( {\tilde{D}^{{ - \frac{1}{2}}} \tilde{A}\tilde{D}^{{ - \frac{1}{2}}} H^{\left( l \right)} W^{\left( l \right)} } \right)$$

Here, *H*^(*l*)^ represents the input features at layer l in the GCN. *H*^(*l*+1)^ signifies the output features at layer l + 1 after the convolution operation. *σ* is the activation function (commonly ReLU or Leaky ReLU). *W*^(*l*)^ is the learnable weight matrix at layer. $\tilde{A} = A + I_{n}$ represents the adjacency matrix of the graph, where *A* is the original adjacency matrix, and *I*_*n*_ is the identity matrix. $\tilde{D}$ is the diagonal node degree matrix of $\tilde{A}$.

### Node attentions in graph transformer module

Recently, the Transformer model has extended its application beyond the field of natural language processing to include a wide range of tasks, including link prediction. In the information integration module, we have incorporated both the graph transformer [38–41] and GCN, thereby enhancing the model's flexibility and performance. The two fundamental components of the Transformer are the dot-product attention mechanism and the feedforward network, playing crucial roles in link prediction tasks.

The graph attention formula is:

6 $$h_{i}^{{\left( {l + 1} \right)}} = \mathop \sum \limits_{j = 1}^{n} \alpha_{ij}^{\left( l \right)} W^{\left( l \right)} h_{j}^{\left( l \right)}$$

where $h_{j}^{\left( l \right)}$ is the* i*-th node’s feature vector in layer* l*, *W*^(*l*)^ is the layer’s weight matrix, n is the graph size, and $\alpha_{ij}^{\left( l \right)}$ is the attention weight between nodes *i* and *j* in layer *l*, computed by

7 $$\alpha_{ij}^{\left( l \right)} = \frac{{\exp \left( {e_{ij}^{\left( l \right)} } \right)}}{{\mathop \sum \nolimits_{k = 1}^{n} \exp \left( {e_{ik}^{\left( l \right)} } \right)}}$$

where $e_{ij}^{\left( l \right)}$ is the similarity between nodes *i* and* j* in layer *l*, computed by:

8 $$e_{ij}^{\left( l \right)} = a^{\left( l \right)} \left( {W^{\left( l \right)} h_{i}^{\left( l \right)} ,W^{\left( l \right)} h_{j}^{\left( l \right)} } \right)$$

where *a*^(*l*)^ is a differentiable similarity function in layer *l*, such as dot product, bilinear, multilayer perceptron, etc.

*Layer-wise transformation* At each layer of the Graph Transformer, the hidden states of nodes are updated using multi-head self-attention and feedforward neural networks. The transformation can be summarized as [42]:

9 $$h_{i}^{\left( l \right)} = {\text{MultiHeadAttention}}\left( {h_{i}^{{\left( {l - 1} \right)}} } \right) + {\text{FeedForward}}\left( {h_{i}^{{\left( {l - 1} \right)}} } \right)$$

*Aggregation across heads* The outputs from multiple attention heads are aggregated to obtain the final node representations:

10 $$h_{i}^{\left( l \right)} = {\text{concat}}\left( {h_{i}^{{\left( {l,1} \right)}} ,h_{i}^{{\left( {l,2} \right)}} , \ldots ,h_{i}^{{\left( {l,H} \right)}} } \right)$$

where *H* represents the number of attention heads.

### Overview of the proposed WMAGT model

As shown in Fig. 1, this section will delve into the detailed description of the model, delineating its architecture, methodologies employed, and the intricate components contributing to its predictive capability.

### Graph representation learning with mixed aggregation parameters

In the context of learning node neighborhood information, a hybrid approach is employed utilizing mixed parameters, integrating two distinct graph convolution operations to acquire meaningful representations of graph data. The fundamental idea of hybrid parameters involves a weighted combination of the outputs of two graph convolution operations, thereby generating the final node features.

11 $$out = \beta \cdot {\text{ReLU}}\left( {{\text{Pool}}\left( {XW,A} \right)} \right) + \alpha \cdot {\text{ReLU}}\left( {\tilde{D}^{{ - \frac{1}{2}}} \tilde{A}\tilde{D}^{{ - \frac{1}{2}}} XW} \right)$$

here, *α* and *β* control the relative influence of the two aggregation methods. The Rectified Linear Unit function (ReLU) [43] is employed as activation function, while *Pool* represents a customized pooling operation involving specific manipulations of the adjacency matrix. This process involves the product of the adjacency matrix and node feature matrix, square operations, and some matrix operations. The entire operation can be expressed mathematically as follows: ${\text{Z}} = ({\text{A}} \cdot {\text{XW}})^{2} - \left( {{\text{A}}^{2} \odot {\text{XW}}^{2} } \right)$. Here, A is the adjacency matrix of the graph, XW is the node feature matrix, and Z represents the new node representation matrix obtained after the graph pooling operation.

This operation introduces additional information into the graph structure, aiming to better capture the relationships between nodes. In general, the introduction of hybrid parameters imparts adaptability to the model, allowing it to determine the relative contributions of different graph convolution operations during the learning process and thus better adapt to diverse graph structures.

### Computing weighted matrices for drug and disease nodes

To compute the weighted matrices for drugs and diseases, we utilize the input feature matrix $X \in {\mathbb{R}}^{N \times d}$, where *N* represents the number of nodes and *d* denotes the feature dimension. The weight matrix, denoted as $W \in {\mathbb{R}}^{{d \times d^{\prime}}}$, corresponds to the output feature dimension *d*′. The graph's adjacency matrix, $A \in \{ 0,1\}^{N \times N}$, demonstrates connections between nodes in a symmetric matrix form. The weighted feature matrix is obtained from this process, which can be represented as:

12 $$XW = X \cdot W$$

the iterative graph convolution concludes with the normalization of the resulting feature matrix using a normalization matrix represented as:

13 $$out = norm \cdot out$$

Furthermore, an element-wise addition of a bias term is performed to further refine the output matrix.

14 $$out = out + self.{\text{bias}}$$

### Compute the element-wise product of drug and disease embeddings

Given an input drug embedding matrix as $D \in {\mathbb{R}}^{{N_{drug} \times {\text{d}}}}$, and a Disease Embedding matrix as $E \in {\mathbb{R}}^{{N_{disease} { } \times d}}$, where *N*_*drug*_ and *N*_*disease*_ represent the quantities of drugs and diseases respectively, and d represents the embedding dimension.

Projection of Drug and Disease via Linear Mapping:

15 $$y = x_{1} \cdot P_{1}$$

16 $$y = { }x_{2} \cdot P_{2}$$

this involves computing the element-wise product of drug and disease embeddings. For instance, let *D*_*ij*_ represent the row and *j*th column element of matrix *D*, and *E*_*ij*_ represent the *i*th row and *j*th column element of matrix *E*. The element-wise product *P* can be obtained as: $P_{ij} = D_{ij} \times E_{ij}$. The resulting matrix *P* captures the element-wise products of the drug and disease embeddings. This process facilitates the exploration of interactions between drugs and diseases within a feature space defined by their embeddings.

To normalize the association matrix P, L_2_ normalization is applied row-wise post element-wise product computation. Each row’s L_2_ norm is computed, and its elements are divided by this norm, ensuring unit L_2_ norm per row. The normalization formula is: $P_{{{\text{norm}}ij}} = \frac{{P_{ij} }}{{\sqrt {{\Sigma }_{k = 1}^{N} P_{ik}^{2} } }}$, Where N denotes the column count, and the summation extends over the row’s elements. This yields a matrix *P*_norm*ij*_ with standardized rows, enhancing association representation and minimizing dataset bias.

### Neural collaborative filtering for drug and disease expression

Neural Collaborative Filtering (NCF) [44] is a neural network-based collaborative filtering algorithm designed for learning relationships between users and items for recommendation purposes. The implementation of NCF in our model involves key components: Neighbor Embedding Process, defines the neighbor embedding process, and integrating information from neighbors of drugs and diseases to better capture relationships between nodes. Interaction Embedding Process, defines the interaction information between drugs and diseases. This section mainly involves calculating interaction embedding through element-wise multiplication and normalization operations. Decoding Process, defines the decoder, transforming embedded node representations into final prediction scores. This process primarily involves linear transformations and non-linear activation functions.

In summary, in the forward method of the model, node embedding representations are first obtained through processes such as neighbor embedding and interaction embedding. Subsequently, the decoder yields the final prediction scores. The core idea of Neural Collaborative Filtering involves learning implicit relationships between drugs and diseases through processes such as embedding, neighbor embedding, interaction embedding, and decoding.

### Neighbor-weighted interaction decoding

In this module, the descriptions of drug–disease associations, drug proximity, and disease proximity are amalgamated into a unified vector $\tilde{h}_{r,d}$ using the concatenation operation ⊕ , defined as:

17 $$\tilde{h}_{r,d} = h_{r,d} \oplus h_{r,d} \oplus \tilde{h}_{r} \oplus \tilde{h}_{d}$$

Here, the operator ⊕ signifies concatenation, facilitating the formation of an encompassing representation that merges established associations with contextual information drawn from drug and disease proximities.

Subsequently, linear transformations and ReLU activation were utilized in processing the hidden layers. In each hidden layer *i* where *i* ranges from 1 to the length of *hidden_dims*, the use of linear transformations and ReLU activation generated *z*_*i*_. Afterwards, at the output layer, linear transformations and Sigmoid activation were applied to handle the outputs from the hidden layer $z_{{{\text{len}}\left( {{\text{hidden}}\_{\text{dims}}} \right)}}$, producing the output *y*. The overall model output *Y* can be interpreted as probabilities for specific categories.

### MLP-based prediction

The introduction of Multilayer Perceptron (MLP) is motivated by its ability to capture intricate nonlinear relationships, extract advanced features, manage sparse data, and exhibit a flexible architecture adaptable to various data traits. Within drug–disease association studies, integrating MLP aims to enhance the accurate prediction and interpretation of complex drug–disease associations, thereby providing deeper insights into correlation studies within the pharmaceutical domain.

Forward propagation in an MLP involves multiple layers, each with numerous neurons. Assuming inputs *X*, *H* neurons in the hidden layer, output *Y*, weight parameters *W*, and biases *b*, the forward propagation can be represented as:

18 $$Y = \sigma \left( {W_{{{\text{output}}}} \cdot \sigma \left( {W_{{{\text{hidden}}}} \cdot X + b_{{{\text{hidden}}}} } \right) + b_{{{\text{output}}}} } \right)$$

### Parameters setting

Hyperparameter settings are crucial for fine-tuning the neural collaborative filtering model, covering dimensions like node embedding, neighbor embedding, and decoder hidden layers. Specifically, the node embedding dimension is set at 64, the neighbor embedding dimension at 32, and the decoder hidden layer dimension is specified as (64, 32). The learning rate is set to 5e − 4, and the dropout rate is 0.3. Additionally, a comprehensive set of loss functions is utilized, encompassing binary cross-entropy loss, focal loss, mean squared error loss, and ranking loss. For the focal loss, parameters are configured with α set to 0.5 and γ set to 2.0. The graph transformer network parameter is defined as λ = 0.8. Throughout the training process, a holistic consideration of these loss functions is conducted, aiming to comprehensively optimize the model. These configurations are designed to strike a balance between model complexity and performance, ensuring optimal predictive outcomes across diverse facets.

Loss Function Formula:

19 $${\text{Focal Loss}} = - \alpha \cdot (1 - p)^{\gamma } \cdot {\text{log}}\left( p \right)$$

Here, *α* controls the balance of weights between positive and negative samples, and γ regulates the focus of the focal loss. We use the Adam [45] optimizer to update model parameters, ensuring efficient training. A cyclic learning rate scheduler dynamically adjusts the learning rate, enhancing training effectiveness. Additionally, the model incorporates two graph neural network layers (Graph Transformer and Graph Convolution Network), employing different neighbor sampling quantities during training.

### Evaluation metrics

We adopted six widely used indicators to measure the predictive performance of the proposed model, including accuracy (Acc), Area Under the Precision-Recall Curve (AUPR), Area Under the Receiver Operating Characteristic Curve (AUC), F1 score, Precision and Recall. Since AUPR and F1 are more sensitive to severe imbalances data. Micro metrics are used for AUPR and AUC, while macro metrics are used for other measurements. The definitions of these indicators can be described as follows:

20 $$Acc = \frac{TP + TN}{{TN + TP + FN + FP}}$$

21 $$precision = \frac{TP}{{TP + FP}}$$

22 $$Recall = \frac{TP}{{TP + FN }}$$

23 $$F1 = \frac{2 \times precision \times recall}{{precision + recall}}$$

where the *TN*, *PN*, *FN* and *FP* denote the number of correctly predicted positive and negative samples, wrongly predicted positive and negative samples, respectively. In addition, we use the *Micro* mode to calculate AUC and Recall, which treats each element of the label indicator matrix as a label. In contrast, F1 calculates each label in a *Macro* mode and finds their unweighted average.

### Baseline methods

NIMCGCN [20]. This study introduces a novel approach named Neural Inductive Matrix Completion with Graph Convolutional Network (NIMCGCN), amalgamating Graph Convolutional Networks (GCNs) and Neural Inductive Matrix Completion (NIMC) models to forecast the association between miRNAs and diseases. By optimizing parameters through supervised learning and demonstrating its superiority in prediction accuracy and forecasting new diseases during experimental validation, the method serves as an effective computational tool for swiftly identifying disease-associated miRNAs.

DRWBNCF [22]. This study introduces a new method called DRWBNCF for drug repositioning, addressing limitations of traditional latent factor models. Leveraging deep learning techniques and a heterogeneous network framework, DRWBNCF infers potential drugs for diseases. By amalgamating drug–disease association information and drug–disease similarity networks, employing a weighted bilinear graph convolution operation, and utilizing a multi-layer perceptron combined with α-balanced focal loss function and graph regularization, DRWBNCF demonstrates effectiveness in predicting unknown drug–disease associations.

Ghasemian ‘s model [24]. Ghasemian et al. employed a meta-learning approach within network analysis to devise a stacked model, amalgamating various sophisticated prediction algorithms. This approach successfully mitigated the variations observed in link prediction across diverse domains of networks.

VAGE [21]. Variational Graph Auto-Encoders (VGAE) is a graph neural network model built upon the framework of Variational Autoencoders (VAE). VGAE integrates the encoder-decoder structure of VAE, modeling node features into latent space distributions and reconstructing them back to the original feature space. Key features include probabilistic modeling, representing node embeddings as Gaussian distributions using reparameterization techniques and KL divergence, while also considering graph structure through graph convolutional networks (GCNs) to efficiently capture local structural information.

DRGBCN [23]. DRGBCN presents an approach that utilizes bilinear attention networks and local interactive learning to improve performance in drug repositioning tasks. Significant performance gains are achieved by emphasizing local association and deep learning applications in the medical domain.

## Results and discussion

### Comparison of WMAGT and state-of-the-art methods under tenfold cross-validation

**Fig. 2:**
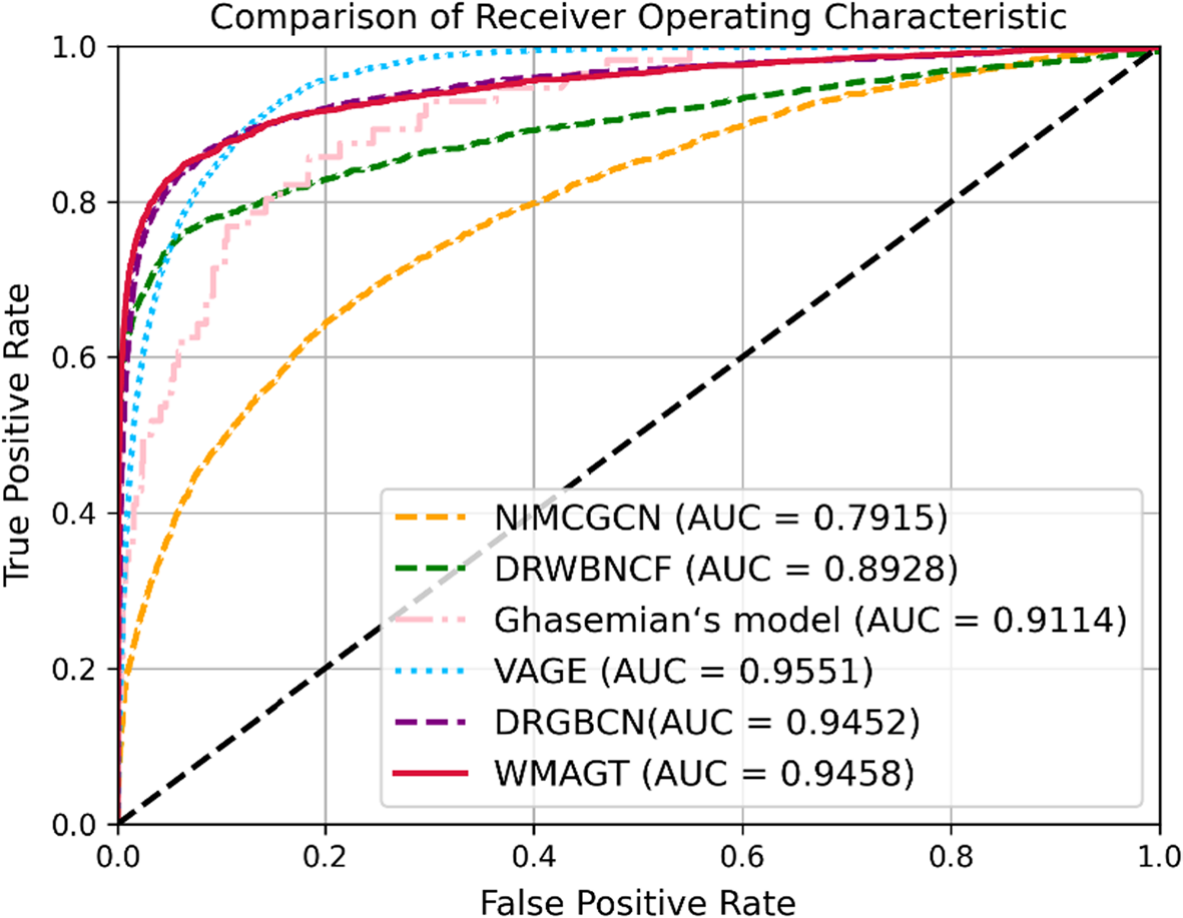
The performance of WMAGT and other compared methods under tenfold cross-validation on Cdataset

**Fig. 3:**
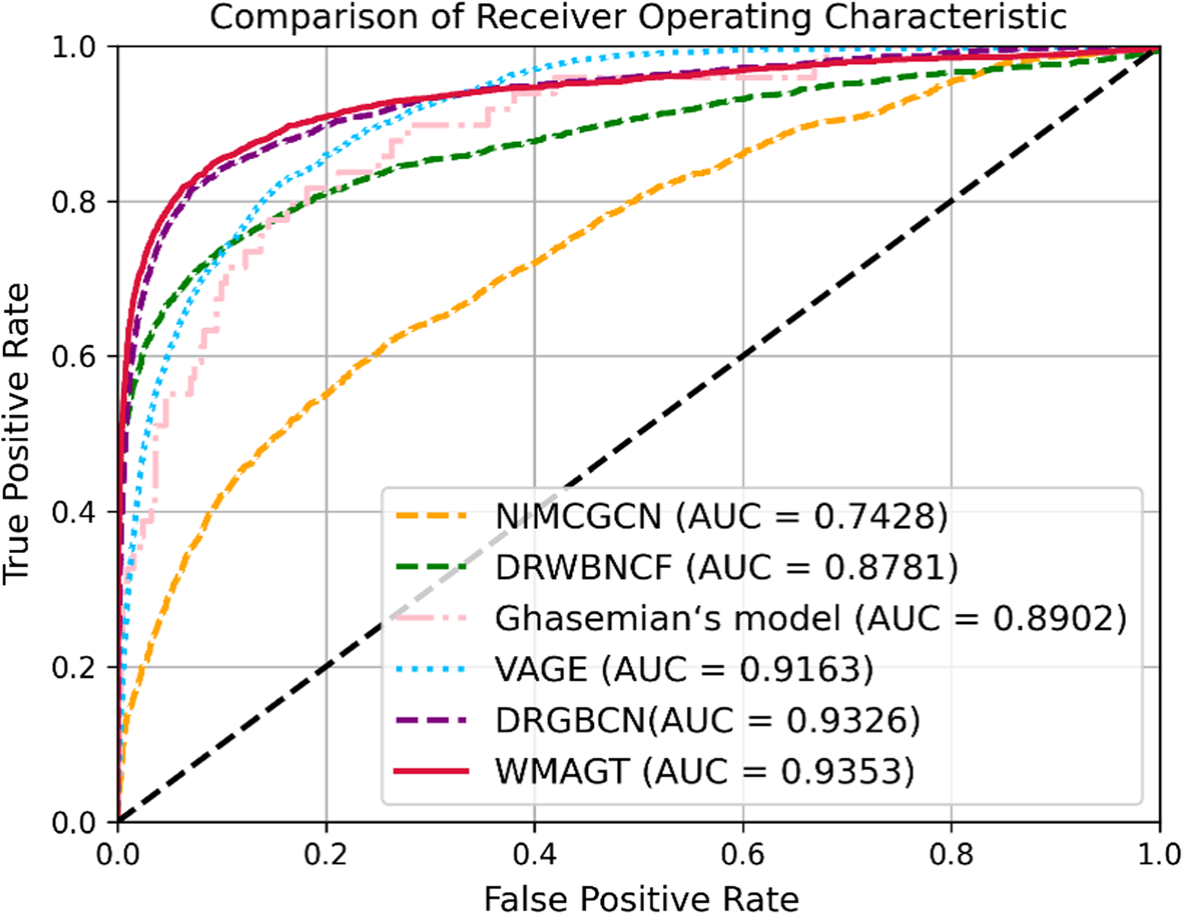
The performance of WMAGT and other compared methods under tenfold cross-validation on Fdataset

**Fig. 4:**
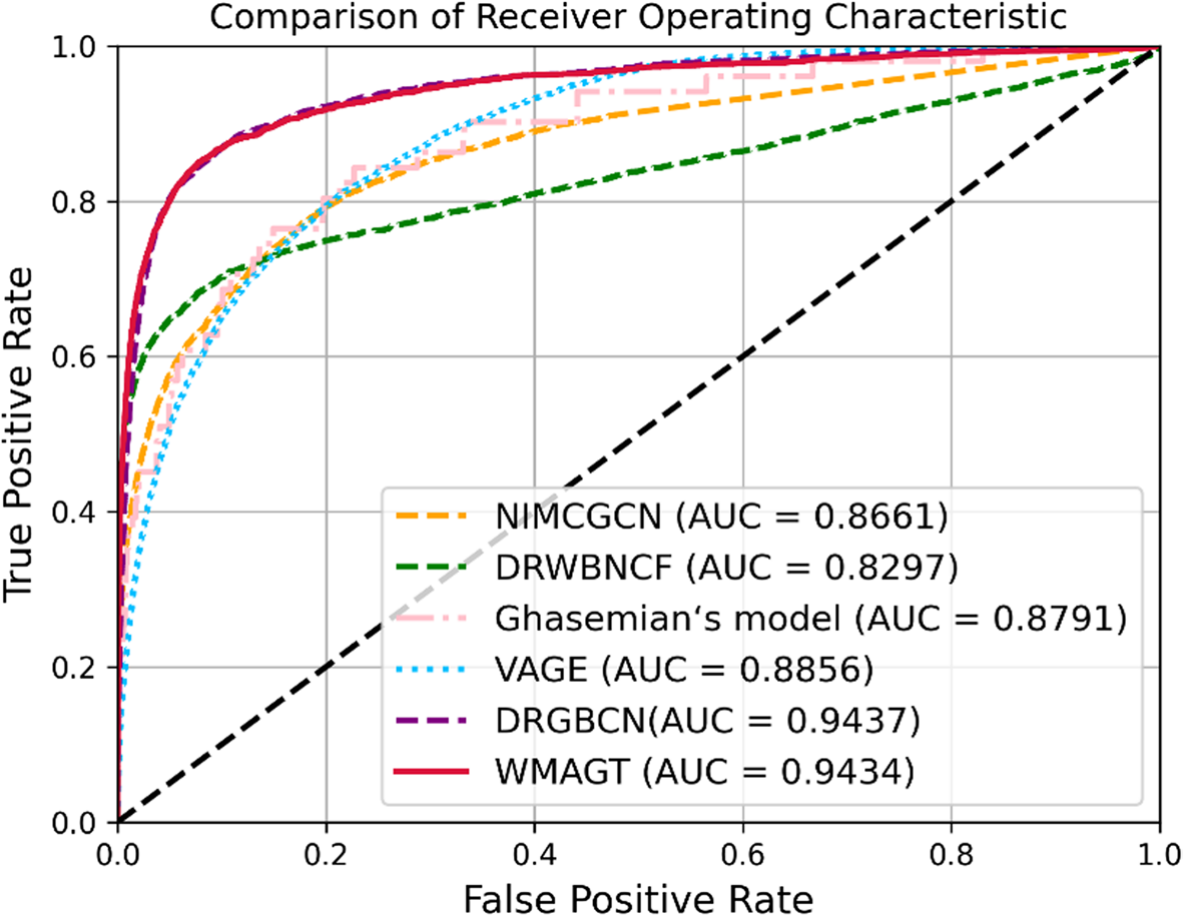
The performance of WMAGT and other compared methods under tenfold cross-validation on LRSSL

To evaluate the performance of the WMAGT model, we conducted extensive experiments on three benchmark datasets, comparing WMAGT with five state-of-the-art methods under tenfold cross-validation. Table 2, Figs. 2, 3 and 4 present the performance of comparison models, including NIMCGCN, DRWBNCF, Ghasemian's model, VAGE, WMAGT and DRGBCN, across different datasets (Fdataset, Cdataset, LRSSL).

**Table 2:** Performance of WMAGT and other compared methods on three benchmark datasets

Datasets	NIMCGCN	DRWBNCF	Ghasemian’s model	VAGE	DRGBCN	WMAGT
*AUROC*						
Fdataset	0.7428 ± 0.0276	0.8781 ± 0.0192	0.8902 ± 0.0328	0.9163 ± 0.1052	0.9326 ± 0.013	**0.9353 ± 0.012**
Cdataset	0.7928 ± 0.0248	0.8928 ± 0.0154	0.9114 ± 0.0292	**0.9551 ± 0.0842**	0.9454 ± 0.0091	0.9458 ± 0.0114
LRSSL	0.8661 ± 0.0165	0.8297 ± 0.0161	0.8791 ± 0.0359	0.8856 ± 0.0536	**0.9437 ± 0.005**	0.9434 ± 0.0083
Average	0.8006	0.8669	0.8936	0.9189	0.9405	**0.9415**
*AUPR*						
Fdataset	0.0558 ± 0.0106	0.4638 ± 0.0548	0.4046 ± 0.0683	0.0589 ± 0.0429	0.4087 ± 0.0281	**0.5231 ± 0.0487**
Cdataset	0.0751 ± 0.0138	0.5801 ± 0.0332	0.4881 ± 0.1047	0.0608 ± 0.0355	0.4517 ± 0.0423	**0.6 ± 0.0429**
LRSSL	0.1807 ± 0.0204	0.4033 ± 0.0201	**0.4925 ± 0.1166**	0.0381 ± 0.0144	0.2558 ± 0.033	0.3651 ± 0.026
Average	0.1039	0.4824	0.4617	0.0526	0.3721	**0.4961**

The bold indicates the best performing method on each metric

Notably, while VAGE achieved a slightly higher AUROC of 0.9551 on the Cdataset compared to our proposed model's 0.9458, and DRGBCN attained an AUROC of 0.9437 compared to our proposed model's 0.9434 on the LRSSL dataset, WMAGT model consistently demonstrated the highest average AUROC value 0.9415 across all datasets. Despite DRGBCN exhibiting a secondary performance in AUROC 0.9405, WMAGT surpasses DRGBCN by more than 10% in terms of AUPR on each dataset. VAGE, Ghasemian's model, DRWBNCF, and NIMCGCN models secured the third, fourth, fifth and sixth positions with AUROC values of 0.9189, 0.8936, 0.8669, 0.8006, respectively. AUPR, particularly sensitive to imbalanced datasets of positive and negative samples, serves as an indispensable evaluation metric. The WMAGT model excelled in AUPR performance, boasting the highest average AUPR value 0.4961, indicating its robust performance under the precision-recall curve. In contrast, the AUPR performances of the other five models were as follows: NIMCGCN 0.1039, DRWBNCF 0.4824, Ghasemian's model 0.4617, VAGE 0.0526 and DRGBCN 0.3721. Average performance is a crucial indicator for assessing the overall effectiveness of models. In this regard, WMAGT demonstrated relatively superior average performance in both AUROC and AUPR, highlighting its effectiveness across various datasets. After statistical testing and analysis, the proposed WMAGT shows significant performance improvement compared to compared models.

### Ablation study

In this section, we delve deeply into the far-reaching impacts of two pivotal modules on our experimental framework:

- 'w/o Transformer': Our investigation goes beyond, scrutinizing the specific effects of excluding the transformer mechanism on model performance. This involves understanding how the model handles information, learns representations, and ultimately predicts drug–disease relationships.
- 'w/o NCF': Further discourse is dedicated to the model's performance in the absence of collaborative filtering. This decision plays a crucial role in determining the model's effectiveness in handling user-item associations, particularly in our specific application scenario.

**Fig. 5:**
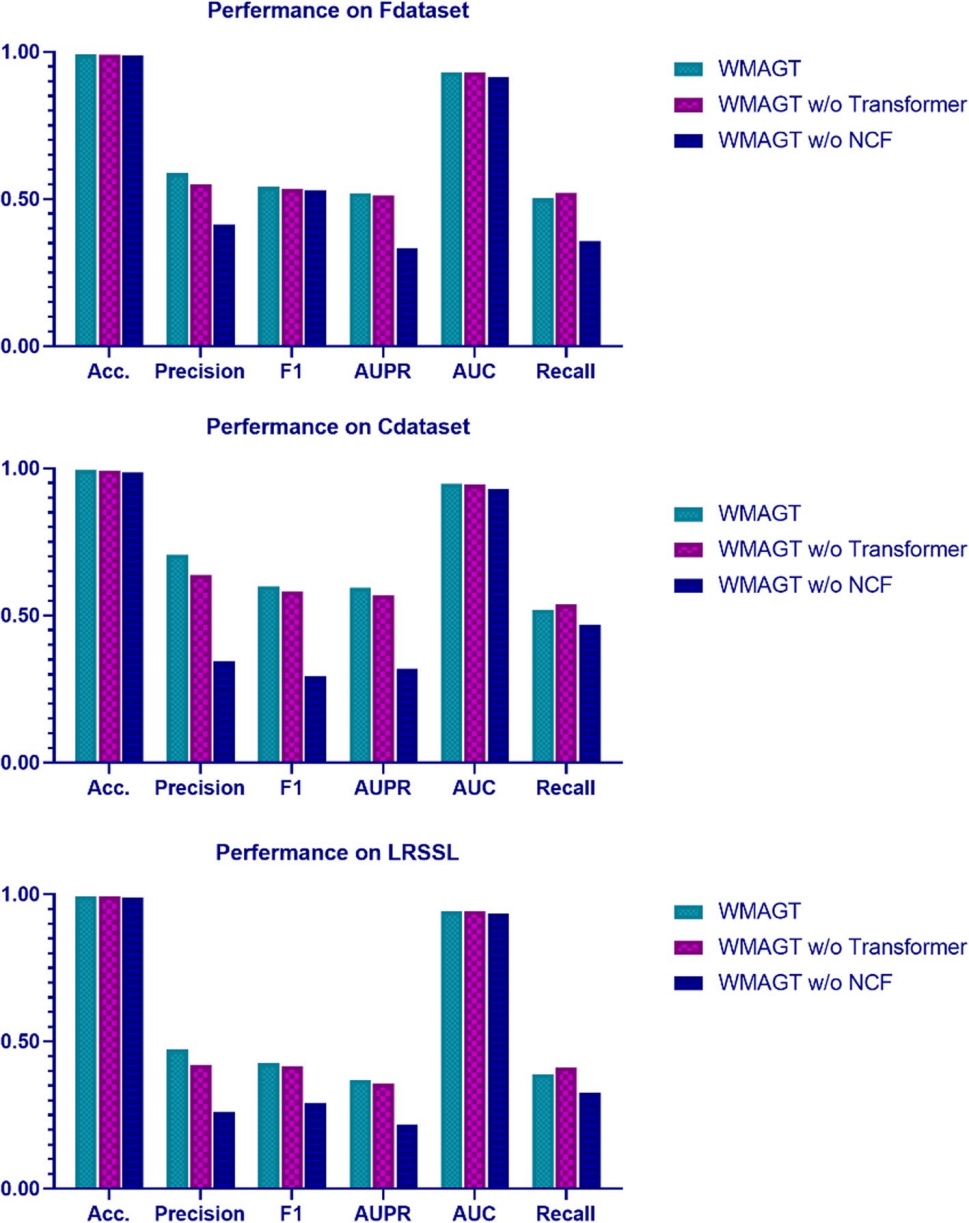
The performance of WMAGT and other variants under tenfold cross-validation on three benchmark datasets

In WMAGT model, we employed a simplified approach, omitting the steps of neighbor embedding and interaction embedding, directly feeding the node representations obtained from the graph convolution module into the decoder. The rationale behind this decision and its implications on model performance necessitate a broader contextual understanding. The results of the ablation study in Fig. 5 showcase the consequences of these decisions. Notably, both the transformer module and the NCF module contribute significantly to enhancing the performance in predicting drug–disease relationships, with the NCF module being particularly noteworthy. This indicates that, when considering multiple embeddings and the transformer comprehensively, the model can more accurately capture latent relationships between drugs and diseases, thereby improving the predictive accuracy of drug–disease relationships. This finding provides profound insights for future model optimization and further research endeavors.

### Case study

To assess the practical applicability of WMAGT, a case study was conducted with the aim of predicting drug candidates for Parkinson's disease. Specifically, the model was trained using all known drug–disease associations in the F dataset, and a descending order ranking was performed after obtaining the probabilities of all drug–disease associations. In this process, the top 10 drug candidates associated with Parkinson's disease were selected for in-depth investigation. Parkinson's disease is a chronic neurological disorder typically characterized by symptoms such as movement disorders, muscle stiffness, and tremors. The primary cause of this disease is the loss of dopamine-producing neurons in the brain, where dopamine functions as a neurotransmitter controlling movement. Currently, the treatment focus for Parkinson's disease primarily revolves around alleviating symptoms, and the exploration of new drug treatment directions has been a crucial area of scientific research. Encouragingly, the relevance of seven of these drugs was further confirmed by additional literature, as depicted in Table 3. This discovery not only enhances the reliability of our model but also indicates that WMAGT successfully identifies potential drug–disease pairs by learning multi-source information about drugs and diseases.

**Table 3:** The top 10 WMAGT-predicted candidate drugs for Parkinson's disease

Rank	Candidate drugs (DrugBank IDs)	Evidence (PMID)
1	Bupivacaine (DB00297)	NA
2	Hydromorphone(DB00327)	NA
3	Clotrimazole(DB00257)	12679339
4	Methylphenidate(DB00422)	18978488
5	Modafinil(DB00745)	12489899
6	Atenolol(DB00335)	NA
7	Ropinirole(DB00268)	9270567
8	Metformin(DB00331)	32854858
9	Guanidine(DB00536)	9548197
10	Olanzapine (DB00334)	11815682

## Conclusions

In this study, we propose a heterogenous information graph-based method for predicting drug–disease associations, named WMAGT. WMAGT innovatively integrates Graph Transformer Networks and Neural Collaborative Filtering, with a core improvement lying in the deep aggregation of local neighbors around nodes to enhance traditional graph convolution operations. Simultaneously, the model autonomously learns to select weights for different types of convolutional networks, resulting in a significant performance improvement compared to a singular graph convolution network. Extensive experiments were conducted to thoroughly assess the performance and robustness of WMAGT. WMAGT exhibited superior performance on three benchmark datasets, better than other compared state-of-the-art models. Ablation studies further verified the importance of different modules introduced in the proposed framework. In addition, the case study show that WMAGT has high practical predictive power, e.g., in Parkinson's potential drug mining, 7 of the top 10 drugs we predicted have been relevantly demonstrated. This study not only introduces methodological refinements but also substantiates their feasibility and superiority through rigorous experimentation and empirical validation. It’s anticipated that these results can serve as valuable references for fostering further drug development and disease treatment.

## Data Availability

The code and datasets are freely available at: https://github.com/ShiHHe/WMAGT.
